# Exploring the Health and Economic Burden Among Truck Drivers in Australia: A Health Economic Modelling Study

**DOI:** 10.1007/s10926-022-10081-4

**Published:** 2022-11-10

**Authors:** Peter Lee, Ting Xia, Ella Zomer, Caryn van Vreden, Elizabeth Pritchard, Sharon Newnam, Alex Collie, Ross Iles, Zanfina Ademi

**Affiliations:** 1 School of Public Health and Preventive Medicine, Monash University, Melbourne, Australia; 2 School of Health and Social Development, Deakin University, 221 Burwood Highway, Melbourne, VIC 3125 Australia; 3 Monash Addiction Research Centre, Monash University, Melbourne, Australia; 4 Monash University Accident Research Centre, Melbourne, Australia; 5 Centre for Medicine Use and Safety, Faculty of Pharmacy and Pharmaceutical Sciences, Monash University, Melbourne, Australia

**Keywords:** Truck driver, Transport worker, Productivity-adjusted life year, Cost burden, Disease burden

## Abstract

*Background* The transport and logistics industry contributes to a significant proportion of the Australian economy. However, few studies have explored the economic and clinical burden attributed to poor truck driver health. We therefore estimated the work-related mortality burden among truck drivers over a 10-year period. *Methods* Dynamic life table modelling was used to simulate the follow-up of the Australian male working-age population (aged 15–65 years) over a 10-year period of follow-up (2021–2030). The model estimated the number of deaths occurring among the Australian working population, as well as deaths occurring for male truck drivers. Data from the *Driving Health* study and other published sources were used to inform work-related mortality and associated productivity loss, hospitalisations and medication costs, patient utilities and the value of statistical life year (VoSLY). All outcomes were discounted by 5% per annum. *Results* Over 10 years, poor truck driver health was associated with a loss of 21,173 years of life lived (discounted), or 18,294 QALYs (discounted). Healthcare costs amounted to AU$485 million (discounted) over this period. From a broader, societal perspective, a total cost of AU$2.6 billion (discounted) in lost productivity and AU$4.7 billion in lost years of life was estimated over a 10-year period. Scenario analyses supported the robustness of our findings. *Conclusions* The health and economic consequences of poor driver health are significant, and highlight the need for interventions to reduce the burden of work-related injury or disease for truck drivers and other transport workers.

**Supplementary Information:**

The online version contains supplementary material available at 10.1007/s10926-022-10081-4.

## Introduction

The transport and logistics industry contributes to a considerable proportion of the Australian economy. Approximately 380,000 workers were employed within the industry in 2020, and an estimated 7.4% (AU$122.3 billion, dollars) of gross domestic product (GDP) was contributed to Australia by the industry in the year 2015–2016 [1, 2]. The disparity in health outcomes between workers in the transport and logistics industry, and workers in other industries is well-established locally and internationally [3]. A recent report found that the incidence of work-related injury or disease among truck drivers was considerably greater than for workers in other, non-transport industries (incidence rate ratio (IRR): 2.39, 95% confidence interval (CI) 2.11–2.69) [4, 5]. This was driven largely by claims for musculoskeletal and trauma-related injuries [4]. Working time lost due to injury or illness was correspondingly high, with the median duration of time loss ranging from three to four weeks among 60% of truck drivers with accepted workers’ compensation claims [4]. Additionally, there is a high prevalence of comorbidities, including cardiovascular disease, obesity, and hypertension among workers which contribute to the considerable mortality burden in the transport industry [3, 6, 7]. This has been attributed to long working hours, poor access to healthcare, exposure to physical and mental stress and other behaviours and occupational characteristics which contribute to poor health [7–10].

*Driving Health* is a multi-stage project aiming to profile the health and wellbeing of Australian truck drivers, and develop ready to implement strategies to help drivers to be healthy and stay healthy at work [4, 6, 8, 9, 11–14]. To date the project explored the health burden, as well as trends in health service usage attributed to truck drivers and other transport workers in Australia [4, 13]. While there is clear evidence of a health gap, interventions trialled in the transport industry, to date, appear focused on dietary interventions [15], personalised apps to manage health and wellbeing and other interventions focused on improving the capacity of the individual to cope with the demands of the job. The findings from the Driving Health project suggest that individually focused interventions should not be the only strategy to support driver health, and that a system-wide approach which incorporates other stakeholders (including regulators and employers) is required to address such multifactorial problems [14]. To date, there are limited studies exploring the long-term economic burden attributed to poor health among transport workers [15–19], and a clear economic argument for improving driver health would provide a compelling case for a collective effort across industry. The high disease and productivity burden attributed to poor health among transport workers is likely associated with considerable economic impacts and warrants further study. The Driving Health project provides an opportunity to combine data from an administrative level, purposively collected data from truck drivers and population level data to guide economic modelling [1, 2, 4–7, 14, 20]. In this present analysis we estimated the future burden of work-related mortality among truck drivers in Australia over a ten year time horizon, as well as explored the economic burden of lost productivity and healthcare usage due to work-related mortality.

## Methods

### Economic Model

Dynamic life table modelling was used to simulate the follow-up of the Australian male working-age population (aged 15–65 years) over a 10-year period of follow-up (2021–2030) [21, 22]. The model estimated the number of deaths occurring among the Australian working population, as well as deaths occurring for male truck drivers. Deaths were assumed to occur halfway through each annual cycle. As the model was dynamic in nature, the population was updated in each cycle through considering deaths and net inward migration. As the model explored the impact of truck driver health on the Australian working population (aged > 15 years) over a 10-year period, births were not captured as part of the dynamic model. Additional details pertaining to the methods used in dynamic health economic modelling have been published elsewhere [21, 22]. The analysis considered both a healthcare and societal perspective, and a 5% annual discount rate was applied to all cost and health outcomes from 2022 onwards [23].

The model was constructed using Microsoft Excel^®^ (Microsoft Corporation, Redmond, WA, US) and the @Risk^®^ extension.

### Model Inputs

#### Population Demographics and Mortality

Our base case modelled population was profiled on the Australian male working-age population (aged 15–65 years) in 2020, sourced from the Australian Bureau of Statistics (ABS) [24]. This was stratified by age, in single years. The model population was limited to working-aged males, as the transport industry in Australia is predominately male [2, 4]. The 2020 population represented the model population in the year prior to the baseline year of 2021, and was evolved for each age strata through considering deaths and net migration occurring from 2021 onwards. In order to estimate the number of deaths occurring in each annual cycle, the population in each age strata were multiplied by the age-specific risk of all-cause mortality for males. This was estimated using mortality data for the year 2020 sourced from the ABS [20], and the model assumed that the risk of death would remain constant throughout the modelled time horizon (10 years). Data pertaining to net migration within each age strata was also sourced from the ABS, with medium inward migration estimates being used in the economic model [25].

#### Truck Driver Health Burden

The incidence of key outcomes considered in the economic model are presented in Table 1.

**Table 1:** Input parameters used in the economic model

Parameter	Point value (range)	Distribution in the PSA	Source
Health burden			
Mortality		Uniform	*Driving Health* Report #2 [12]
Direct	0.03% (± 30%)		
Indirect^a^	0.17% (± 30%)		
Driver injury		Static	*Driving health* Report #2 [12]
Fractures	0.63% (± 30%)		
Musculoskeletal	4.18% (± 30%)		
Neurological	0.23% (± 30%)		
Mental health	0.09% (± 30%)		
Other traumatic	1.66% (± 30%)		
Other disease claims	0.20% (± 30%)		
Productivity index^b^		Uniform	AHRI [30], Troelstra [31]
General population	0.92 (± 15%)		
Truck drivers	0.86 (± 15%)		
Utilities (mean, SD)^c^		Beta	McCaffrey et al. [27] *Driving Health* Report #6 [11]
General population	0.92 (0.13)		
Truck drivers	0.83 (0.16)		
Costs			
Mortality costs		Gamma	
Direct mortality	$0		Assumption
Indirect mortality	$7087 (± 30%)		AR-DRG[35]
Hospitalisation costs		Gamma	*Driving Health* Report #3 [8]
Fractures	$11,305 (± 30%)		
Musculoskeletal	$7016 (± 30%)		
Neurological	$14,404 (± 30%)		
Mental health	$6768 (± 30%)		
Other traumatic claims	$8610 (± 30%)		
Other disease/claims	$3721 (± 30%)		
Medications costs	$1257 (± 30%)	Gamma	*Driving Health* Report #4 [9]
VoSLY	$222,000 (± 30%)	Uniform	Department of the Prime Minister and Cabinet [36]

*PSA* probabilistic sensitivity analysis, *VoSLY* value of statistical life year

Costs are reported in Australian dollars (AU$) for the year 2021

^a^Derived from all-cause mortality rates among truck drivers and direct truck-related deaths

^b^Derived from estimated absenteeism and presenteeism values from the Australian Human Resources Institute, and Troelstra et al

^c^Normative Australian age and sex-specific values were used to estimate age-specific utility values for truck drivers (see Online Appendix B)

The prevalence of work-related mortality among truck drivers was estimated using data from the *Driving Health* study [12]. Specifically, national workers’ compensation claims data from the National Dataset for Compensation-based Statistics (NDS) for the period of 2004–2015 were used in estimating the incidence of work-related mortality among truck drivers [4]. Separately, the incidence of all-cause mortality among truck drivers was also drawn from life insurance claims data collected as part of the *Driving Health* study for the period of 2004–2017 [4, 6]. The difference between the incidence of work-related mortality, and incidence of all-cause mortality among truck drivers was used to estimate the indirect mortality burden among truck drivers.

A recently published *Driving Health* study explored health services usage using data from the WorkSafe Victoria Compensation Dataset between 2004 and 2013 was used to estimate the incidence of hospitalisations occurring among truck drivers [8, 26]. Seven major categories of injuries were considered, including hospitalisations for fractures, musculoskeletal injuries, neurological or mental health conditions as well as hospitalisations for other traumatic injuries, other diseases, and other claims. Additional details pertaining to these datasets have been published elsewhere [4, 6, 8]. In the base case economic model, it was assumed that the per-capita health burden attributed to truck drivers would remain constant from each year from 2021 to 2030 inclusive. The total disease burden was adjusted to the Australian male working-age population in each year, relative to the baseline population in 2020.

The incidence of work-related and all-cause mortality was used to estimate the risk of truck-driving related deaths occurring annually. Among a population of 353,901 workers in the truck driving industry in 2020, we estimated that 718 deaths (121 direct and 597 indirect) had occurred using death benefit data from the *Driving Health* report [8, 26]. The risk of mortality was estimated through dividing the number of deaths occurring for truck drivers by the Australian male population aged ≥ 15 years in 2020 (10,280,093); that is, 0.007%. This risk was assumed to be the same across each age strata, and was applied to the age-stratified population for each year from 2021 to 2030 inclusive to estimate the number of deaths occurring among truck drivers in each year. A similar approach was used to estimate the risk of hospitalisations accrued over 10 years. That is, in lieu of age-specific incidence data for work-related mortality or hospitalisations, the incidence of mortality was weighted to the proportion of working-aged males across the age strata.

#### Health-Related Quality of Life and Productivity Burden

Utility values and productivity indices considered in the economic model are presented in Table 1.

Utility values reflect a person’s health-related quality of life (HRQoL) based on physical, mental and social health domains estimated using a generic, multi-attribute instrument (MAUI). They lie on a scale from 0 (representing dead) to 1 (full health) with negative values representing health states considered worse than death [27]. A quality-adjusted life year (QALY) incorporates quality of life and survival into a single measure; that is, a QALY reflects individual survival, adjusted for HRQoL and is estimated through multiplying the duration of time spent in a particular health state by the utility score [28]. Based on a survey attributed to 1390 Australian truck drivers, the average HRQoL score for workers in the industry was 0.83 (SD: 0.16), which is considerably lower than Australian norms (mean: 0.91, SD: 0.14) as estimated using the five-level EQ-5D MAUI [11, 27]. This finding of poorer HRQoL was attributed to the poor physical and mental health profile for Australian truck drivers [11]. To better reflect the characteristics of surveyed participants, this utility value was only applied to subjects aged 35–44 years in the model [11]. To subjects aged < 35 years, normative Australian age and sex-specific utilities were used in the model [11, 27]. For subjects aged ≥ 45 years in the truck driving industry, age-related decrements estimated from normative Australian age and sex-specific utilities were applied to the mean utility value of 0.83 to reflect age-related declines in HRQoL. Additional details relating to the estimation of utility values are presented in Online Appendix B. These utility values were multiplied by the estimated years of life lived to estimate QALYs in the model.

**Fig. 1:**
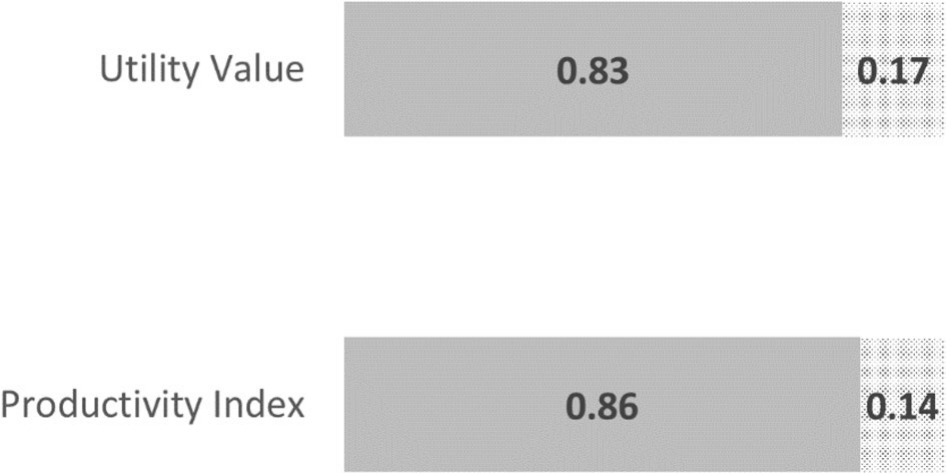
Estimation of PALYs. A utility value of 0.83 among truck drivers indicates a relative reduction in quality of life by 17%. Similarly, a productivity index of 0.86 indicates a relative reduction in productivity of 14%. The estimated number of years of life is multiplied by the productivity index (*utility value*) to estimate PALYs (*QALYs*)

In addition to HRQoL, the model also explored the productivity burden attributed to poor health among Australian truck drivers. Similar to utility values, which reflect quality-of-life, a productivity index reflects the proportional loss in productivity for workers and ranges from 0 (entirely unproductive) to 1 (entirely productive) (Fig. 1).

Loss in productivity can be due to days off work (absenteeism), reduced productivity while at work (presenteeism), as well as reductions in work force participation. The productivity index was estimated through dividing the days worked in a year (that is, without absenteeism and presenteeism) with the maximum working days in a year [29]. The years of life lived estimated in the model were multiplied by the respective productivity index for the male working population, and the truck driving population, to estimate productivity-adjusted life years (PALYs). PALYs are a novel metric, similar to QALYs, which adjust years of life lived by health-related productivity loss rather than adjusting for health-related loss of quality-of-life [29].

In Australia, the maximum working days in a year is 240, which reflects that the population, in general, have four weeks of annual leave per year and work five days per week. On average, absenteeism among the Australian population was 8.8 days per work year based on a report from the Australian Human Resources Institute [30]. Absenteeism among truck drivers varied from two weeks for drivers aged between 15–24 years, to 6.6 weeks for drivers aged ≥ 65 years based on the *Driving Health* study [12]. To estimate the weighted average absenteeism for the modelled truck driver cohort (19.1 days per work year), the age-specific duration of absenteeism estimated in the *Driving Health* study was weighted by the number of male truck drivers in each 10-year age group in 2020, sourced from ABS data [2]. A recently published study of the productivity burden attributed to multimorbidity among an Australian cohort was used to estimate presenteeism for the modelled populations in lieu of presenteeism data specific to the truck-driving population [12, 31]. It was assumed in the model that mean presenteeism among workers outside of the truck driving industry was 11.3 days per work year based on the presenteeism estimate for the general male population in Troelstra et al. [31]. Based on survey data from the *Driving Health* study which found that 29.5% of drivers had three or more diagnosed health conditions (compared with 7.8% in the general population), we assumed that truck drivers presented with two diagnosed health conditions [11, 12, 31]. We estimated a mean presenteeism of 15.2 days per work year among truck drivers due to multimorbidity. These correspond to productivity indices of 0.92 and 0.86 used in the base case economic model (Table 1). Additional details pertaining to the estimation of productivity indices are presented in Online Appendix A.

Labour force participation rates for the male, working-age (15–65 years) population were sourced from the ABS for 2021, stratified by age in single years [2]. To reflect the impact of poor health on labour force participation rates, we assumed that the rate of non-participation in the labour force for truck drivers to be 1.5 times greater than the rate of non-participation for the equivalent age strata in the male, working-age population. This was in lieu of data pertaining to labour force participation rates specific to workers in the truck driving or transport industries; however, the risk of labour force dropout due to early retirement or disability varied between 1.26 (95% CI 1.00–1.58) to 4.46 (95% CI 3.49–5.71) in studies exploring the impact of work in industries with heavy physical load on labour force participation [32, 33]. Labour force participation rates for male working-age (15–65 years) workers in the truck driving industry, and for the total labour force, were applied to population estimates to approximate the number of equivalent full-time (EFT) workers for each age strata in the truck driver cohort, and for the general male working-age population. This was multiplied by the productivity index specific to each cohort, to estimate PALYs.

### Cost Inputs

Key cost inputs used in the economic model are presented in Table 1.

All costs were updated to 2021 values using the Australian Health Price Index [34]. Hospital claims cost data from the *Driving Health* study pertaining to accepted workers’ compensation claims were used to estimate the unit costs of hospitalisations for injuries or diseases related to truck driving. The average hospitalisation cost varied from AU$3,687 [standard deviation (SD): AU$9,196] for hospitalisations claims for other diseases, to AU$11,305 (SD: AU$23,369) for hospitalisations claims for fractures.

The cost of mortality attributed directly to truck driving was assumed to be zero on the basis that no health service use would occur among individuals as a result of direct, work-related fatality. The cost for each indirect mortality event occurring among truck drivers was assumed to be the weighted average of all Australian Refined Diagnosis Related Groups (AR-DRGs) for cardiovascular disease, musculoskeletal or trauma-related injury, and cancer, and their costs for publicly-funded casemix hospitalisations in 2017/18 [35]. In 2017–2018, this was AU$6746 which was updated to a 2021 value of AU$7087 [34]. These were selected on the basis that the majority of death claims among truck drivers were attributed to some form of cardiovascular disease, cancer or external injury [6].

#### Cost of a Year of Life

The value of a statistical life year (VoSLY) represents the cost of each year lived by an Australian, as valued from the societal perspective. The VoSLY was assumed to be AU$220,000 based on the VoSLY estimated by the Australian Government’s Office of Best Practice Regulation in 2021 [36].

#### Cost of Lost Productivity

The cost of lost productivity as a result of poor driver health was estimated by assigning a cost value for each PALY. The unit cost of a PALY for any given year was based on the GDP per EFT estimated for the corresponding year in the economic model (2021–2030 inclusive). Data pertaining to GDP per hour worked were sourced from the ABS, and we assumed a work week was comprised of 40 h and full-time workers had a maximum of 48 weeks worked per working year [37]. For example, the GDP per EFT worker in 2020 was estimated to be AU$192,000, based on a GDP per hour worked of AU$100 × 40 h × 48 weeks. The temporal trend in GDP per hour worked was then used to estimate the GDP per EFT worker for the general population over the modelled period (2021–2030). For reference, the unit cost of a PALY in 2021 was estimated to be AU$196,288 and increased to AU$216,599 in 2030.

#### Modelled Outcomes

The economic model was used to estimate the impact of working in the truck driving industry on years of life lived, QALYs, and PALYs. Years of life lost as a result of working in the truck driving industry, as well as QALYs and PALYs, were estimated only for truck drivers who died prematurely from work-related causes.

### Sensitivity and Scenario Analyses

A variety of deterministic sensitivity analyses were performed through varying key model parameters. These were varied individually while other variables were maintained at base case values to explore the impact of key parameters on modelled outcomes. Key parameters assessed include the time horizon modelled, the assumed number of comorbidities among truck drivers, the costs assumed for hospitalisations and mortality, presenteeism and absenteeism among truck drivers, and the VoSLY assumed (± 30%). The VoSLY was also varied based on recommended values from a recent systematic review of value of statistical life year estimates [38]. We also performed a scenario analysis of a hypothetical intervention designed to reduce the health burden of truck drivers by 2% and by 10%.

The probabilistic sensitivity analysis was undertaken to understand the joint uncertainty of the key model input parameters, such as cost, quality of life and productivity (Table 1). The PSA was performed by running 10,000 iterations of a Monte Carlo simulation. The respective candidate distributions for costs (gamma or uniform), utilities (beta) and productivity indices (beta) were selected as per Briggs et al. [39].

## Results

Over a 10-year period (2021–2030 inclusive), an estimated 6067 lives would be lost due to work-related diseases or injury occurring in the truck driving industry. This corresponded to a loss of 21,173 years of life lived (discounted), or 18,294 QALYs (discounted). Table 2 presents the base case analysis in terms of the overall clinical and cost burden attributed to work-related injuries or diseases occurring among workers in the truck driving industry.

**Table 2:** Results of the economic model

Year	Deaths	YLL	QALYs lost	Healthcare cost	PALYs lost	Cost of PALYs lost	VoSLY lost
2021	581	291	252	$57,592,159	167	$32,726,050	$64,501,097
2022	587	828	719	$55,395,103	477	$94,725,351	$183,817,423
2023	593	1310	1136	$53,259,513	759	$152,384,116	$290,714,800
2024	599	1739	1507	$51,187,641	1014	$205,877,842	$386,106,229
2025	605	2121	1836	$49,176,776	1244	$255,380,813	$470,834,140
2026	610	2458	2126	$47,228,642	1450	$301,056,471	$545,690,201
2027	615	2754	2379	$45,340,439	1635	$343,051,648	$611,404,010
2028	620	3012	2600	$43,524,906	1799	$381,525,163	$668,671,352
2029	625	3235	2789	$41,779,404	1944	$416,657,802	$718,177,709
2030	631	3426	2951	$40,101,384	2071	$448,635,855	$760,589,248
Total	6067	21,173	18,294	$484,585,966	12,560	$2,632,021,110	$4,700,506,209
95% confidence interval	–	$16,144–$26,200	$15,589–20,185	$437,356,016–$532,253,627	$9,571–$15,538	$2,006,051,123–$3,256,521,067	$3,583, 276,125–$5,815,343, 121

*PALY* productivity-adjusted life year, *QALY* quality-adjusted life year, *VoSLY* value of statistical life year, *YLL* years of life lost

Healthcare costs, including hospitalisations and medications costs, amounted to AU$485 million (discounted) over this period. From a broader, societal perspective, a loss of 12,560 PALYs (discounted) was estimated as a result of mortality among truck drivers in Australia. Assuming the cost of each PALY increased over time (AU$196,288 in 2021 to AU$216,599 in 2030), the total cost attributed to productivity loss among workers in the truck driving industry was AU$2.6 billion (discounted) over a 10-year period (2021–2030 inclusive). The VoSLY lost over this period was AU$4.7 billion. Additionally, in Table 2, we present 95% confidence interval values from the PSA for years of life lost, cost, QALYs lost, PALYs and VoSLYs lost.

### Sensitivity and Scenario Analyses

Table 3 presents the results of deterministic sensitivity analyses.

**Table 3:** Results of deterministic sensitivity analyses

Parameter	Deaths	YLL	QALYs lost	Healthcare cost	PALYs Lost	Cost of PALYs Lost	VoSLY lost
Base case	6067	21,173	18,294	$484,585,966	12,560	$2,632,021,110	$4,700,506,209
Mortality (base case: 0.20%)							
Lower 30% (0.15%)	4603	16,063	13,879	$477,847,468	9529	$1,996,797,387	$3,566,062,422
Upper 30% (0.26%)	7650	26,700	23,069	$492,020,357	15,838	$3,319,034,136	$5,927,442,214
Injury risk ^a^							
Lower 30%	–	–	–	$348,071,791	–	–	–
Upper 30%	–	–	–	$621,100,141	–	–	–
Time horizon (base case: 10 years)							
5 years	2965	6288	5449	$266,611,192	3660	$741,094,171	$1,395,973,689
VoSLY (base case: $220,000)							
Lower bound ($155,400)	–	–	–	–	–	–	$3,290,354,346
Upper bound ($288,600) Ananthapavan et al. [38]	–	–	–	–	–	–	$6,110,658,072
Lower bound ($94,096)	–	–	–	–	–	–	$1,992,345,908
Central value ($324,058)	–	–	–	–	–	–	$6,861,427,177
Discounting (base case 5%)							
3%	6067	23,718	20,491	$525,833,089	14,079	$2,953,879,634	$5,265,437,983
Truck driver productivity index (base case: 0.86)							
Lower 5% (0.81)	–	–	–	–	14,055	$2,945,349,174	–
Upper 5% (0.90)	–	–	–	–	13,050	$2,734,787,485	–
Cost of mortality (base case: $7,087)							
Lower 30% ($4,961)	–	–	–	$475,724,352	–	–	–
Upper 30% ($9,214)	–	–	–	$493,447,581	–	–	–
Medications costs (base case: $1,257)							
Lower 30% ($880)	–	–	–	$473,196,231	–	–	–
Upper 30% ($1,633)	–	–	–	$495,975,701	–	–	–
Hospitalisation costs^b^							
Lower 30%	–	–	–	$359,461,526	–	–	–
Upper 30%	–	–	–	$609,710,406	–	–	–
HR for truck driver labour non-participation (base case: 1.5)							
Equivalent labour force participation rates	–	–	–	–	13,027	$2,730,064,236	–
Driver health intervention (base case: no intervention)							
Reduced health burden (2%)	5946	20,750	17,928	$474,897,497	12,309	$2,579,390,505	$4,606,513,334
Reduced health burden (10%)	5460	19,056	16,465	$436,142,295	11,304	$2,368,864,077	$4,230,534,796

*HR* hazard ratio, *PALY* productivity-adjusted life year, *QALY* quality-adjusted life year, *VoSLY* value of statistical life year, *YLL* years of life lost

Costs are reported in Australian dollars (AU$) for the year 2021

^a^The incidence of injuries varied between 0.09% for mental health, and 4.2% for musculoskeletal injury

^b^Costs of hospitalisations varied between $3,721 for hospitalisations for other diseases or claims, to $14,404 for hospitalisations for neurological conditions

Key model drivers include the impact of work-related injury or disease on mortality, and on the costs associated with hospitalisations or medications use. The impact of a hypothetical intervention which reduced the health burden experienced by truck drivers by 2% resulted in savings of approximately AU$10 million in healthcare costs, AU$53 million in lost productivity, and almost AU$94 million in VoSLYs. A reduction in driver health burden of 10%, resulted in savings of over AU$48 million in healthcare costs, AU$263 million in lost productivity, and almost AU$470 million in VoLSYs.

## Discussion

Our study explored the burden of work-related mortality among male truck drivers in Australia over a 10-year period (2021–2030 inclusive). Based on the assumption that the health burden attributed to working as truck drivers remained constant over time, we estimated that 6067 lives and 21,173 years of life lived (discounted) would be lost over this period. The cost burden attributed to early mortality arising from work-related injury and illness in the truck driving industry are correspondingly high, with an estimated AU$485 million (discounted) in healthcare costs, AU$2.6 billion (discounted) in lost productivity and AU$4.7 billion in terms of VoSLY. Hence, the clinical and cost benefits attributed to an intervention which reduces the health burden attributed to working in the truck driving industry is likely considerable. This conclusion is consistent with the results of the *Driving Health* study, which suggests that preventive measures focused on improving driver health behaviours and working conditions are likely to reduce this burden [4, 11, 12, 14]. These findings are also consistent with international research in the US and in Canada which explored factors which contribute to poor driver health [3, 7, 40].

The approach employed in the current study is, to the authors’ knowledge, the first to explore the economic implications of the current state of driver health and wellbeing. A recent meta-review of health and wellbeing interventions targeted at heavy vehicle drivers identified eight studies that have explored financial or economic outcomes of interventions [41]. Other US-based studies have examined the implications of how driver remuneration impacts safety, as well as other factors which impact driver health [42, 43]. Rather than describe the impact of a particular intervention, this current study applied a holistic approach to describe the complex issue of driver health, where one intervention is unlikely to be sufficient to address the issue. Intervention at multiple levels within the transport system is required [41], particularly if the considerable savings in healthcare costs, lost productivity and lost life identified in the scenario analysis are to be realised. The findings of the present study indicate that even modest changes in truck driver health would likely be, at a minimum, cost-effective.

A key strength of our study lies in the use of contemporaneous, nationally-representative data from the ABS and from the *Driving Health* study. Hence, the economic model likely captured the majority of cost and clinical consequences attributable to poor truck driver health, as well as the potential benefits of driver health interventions. Additionally, the use of a dynamic life table model allowed for the consideration of natural changes in population sizes over time, including migration [44]. Hence, in comparison with a closed-cohort economic model which follows the same group of individuals over time, the dynamic nature of the economic model allows for a more realistic estimation of key outcomes of interest through capturing net workforce migration [21, 22, 44, 45]. Furthermore, we considered both the healthcare and broader societal consequences attributed to poor truck driver health through estimating direct healthcare costs and clinical burden, as well as lost productivity (PALYs) and the societal costs associated with mortality (VoSLYs) [21, 22, 44, 46]. Importantly, the considerably greater cost of productivity loss (AU$2.6 billion) relative to direct healthcare costs (AU$485 million) estimated in our model is likely indicative of the morbidity burden attributed to mental health conditions among drivers, which were associated with the greatest duration of time off work [12]. PALYs therefore present a novel health metric in quantifying the economic impact of lost productivity attributed to poor driver health, which may not be adequately captured through more conventional metrics such as QALYs or disability-adjusted life years.

Several limitations to our analyses warrant mention. First, workers’ compensation claims data were used to estimate the incidence of truck driver mortality, as well as the incidence of hospitalisations and medications usage. Hence, cases of injury or disease which were not related to work, or were work-related but were not claimed by workers, were not captured [4, 6, 8, 9, 12–14]. Additionally, health service use among self-employed workers, as well as workers with non-compensable injuries or who made multiple claims for injuries or disease were not captured in the data [4, 6, 8, 9, 12–14]. Our analyses were also limited to the male working-age population in Australia, and only explored the cost and productivity burden attributed to work-related mortality among truck drivers. Additionally, mortality was assumed to be the same across each age strata in lieu of age-specific incidence data for work-related mortality, despite studies demonstrating a greater overall risk of mortality with increasing age among drivers [6]. It is therefore likely that we underestimated the true cost and health burden attributed to workers in the overall transport and logistics industry. Second, in lieu of data specific to the Australian truck driving population, presenteeism estimates from a study exploring the impact of multimorbidity on productivity were used [31], and the derivation of PALYs for truck drivers was based on the standard Australian work week (240 maximum working days). The true productivity burden was likely underestimated for truck drivers, due to the considerable variation in work hours attributed to working in the truck driving industry as well as behavioural factors attributed to working in a predominantly male industry. However, varying the productivity index assumed in the model did not change our estimates considerably. Third, we had applied labour force participation rates and mortality rates for 2020 and 2021 to the model without accounting for temporal changes occurring over 10 years. However, patient labour force and mortality rates have remained relatively stable in the 10-year period prior to the modelled analyses [2, 47]. Lastly, data from the *Driving Health* study were collected prior to the COVID-19 pandemic, which was associated with significant changes to truck driver health as well as to the overall transport industry [48, 49]. Ultimately, our conservative assumptions suggest our model likely underestimated the productivity and clinical burden attributed to the truck driving industry. Nevertheless, our conclusion that there are considerable economic and health losses attributed to truck drivers remains unchanged.

## Conclusions

Despite the importance of the transport and logistics industry to the Australian economy, workers are at a greater risk of work-related injury or disease relative to other industries. Our analyses highlight the health and economic consequences of poor driver health over the forthcoming decade, and highlight the need for interventions to reduce the burden of work-related injury or disease for truck drivers and other transport workers.

## Supplementary Information

Below is the link to the electronic supplementary material.

Supplementary file1 (DOCX 14 kb)

## Data Availability

All data are incorporated into the article and its online supplementary material.
