# Supplementary material for: Exploring the Health and Economic Burden Among Truck Drivers in Australia: A Health Economic Modelling Study
Source: J Occup Rehabil. 2022 Nov 10;33(2):389–98. doi: 10.1007/s10926-022-10081-4 (PMC9648998; doi:10.1007/s10926-022-10081-4)
Supplement: Supplementary file 1 — Supplementary file1 (DOCX 14 kb) [file 10926_2022_10081_MOESM1_ESM.docx]

**Appendix A: Estimation of productivity indices**

**Table A1: Estimation of productivity indices (base case economic model)**

| **Parameter** | **Population** | | **Source** |
| --- | --- | --- | --- |
|  | **Truck drivers** | **Non-Truck drivers** |  |
| Absenteeism (days per work year) | 19.1 | 8.8 days | AHRI report [30]  *Driving Health* Report # 2 [12] |
| Impact of co-morbidities on presenteeism (IRR, 95% CI) | 1.16 (1.04 – 1.30) | | Troelstra et al [31] |
| Assumed number of co-morbidities | 2 | 0 | Assumption |
| Presenteeism (days per work year) | 15.2^a^ | 11.3 | Troelstra et al [31] |
| Productivity Index ^b^ | (240 days – 19.1 days absenteeism – 15.2 days presenteeism)/240 = 0.86 | (240 days – 8.8days absenteeism – 11.3 days presenteeism)/240 = 0.92 | Ademi et al [29] |

AHRI = Australian Human Resources Institute

^a^ Based on the assumption that presenteeism increases by 16% with each increase in the number of comorbidities.

^b‑^Based on methodology described in Ademi et al (2021).

**Appendix B: Estimation of utility values**

**Table B1: Estimation of utility values for the economic model**

| **Parameter** | **Truck drivers** | **General population** | **Source** |
| --- | --- | --- | --- |
| Utility values (Mean (SD)) | 0.83 (0.16) ^a^ | 0.92 (0.13) | McCaffrey et al [27]  *Driving Health* Report #6 [11] |
| Utilities by age group (mean, SD)  15 – 24  25 – 34  35 – 44  45 – 54  55 – 64  65+ | 0.96 (0.07)  0.95 (0.10)  0.93 (0.12)  0.90 (0.16)  0.9 (0.14)  0.87 (1.16) | 0.96  0.95  0.83  0.80  0.80  0.77 | Assumption ^b^ |

^a^ This utility value was only applied to subjects aged 35 – 44 years in the model

^b^ For subjects aged < 35 years, normative Australian age and sex-specific utilities were used in the model, while for subjects aged ≥ 45 years in the truck driving industry, age-related decrements estimated from the normative Australian age and sex-specific utilities were applied to the mean utility value of 0.83 to reflect age-related declines in health-related quality of life.
